# Aneurysmal Bone Cyst of the Distal Femoral Metaphysis in a Three-Year-Old Male Patient Presenting With a Pathologic Fracture: A Case Report

**DOI:** 10.7759/cureus.80903

**Published:** 2025-03-20

**Authors:** Xavier Penda, Serge Tima, Ghislain Aminake, Henry Ndasi

**Affiliations:** 1 Orthopaedics and Trauma, Baptist Hospital Mutengene, Mutengene, CMR

**Keywords:** aneurysmal bone cysts, bone defect, bone reconstruction, curetage, distal femur tumors, induced-membrane technique, marginal resection, masquelet's technique, pediatric orthopedics

## Abstract

This case report discusses the management of a three-year-old male presenting with a pathological fracture of the distal femur due to an aneurysmal bone cyst (ABC). Due to diagnostic uncertainty and limited resources, the treatment involved an en bloc resection followed by the Masquelet technique with a fibular and cancellous graft. While this approach successfully reconstructed the bone defect, the patient developed a progressive valgus deformity and limb length discrepancy, likely due to physeal damage from the resection rather than the reconstruction technique itself. This case highlights the challenges of balancing recurrence risk, functional outcomes, and resource limitations in pediatric ABC treatment. A more physeal-sparing approach, such as curettage with bone grafting, might have mitigated complications while preserving long-term limb function.

## Introduction

Aneurysmal bone cysts (ABCs) are benign but locally aggressive bone lesions characterized by expansile, blood-filled cavities. They commonly affect the metaphyseal regions of long bones, including the femur [1], tibia, and humerus, and often present with pain, swelling, and pathological fractures [2]. Imaging studies, including radiographs and MRI, play a crucial role in diagnosing ABCs, as they typically appear as lytic, well-defined lesions with septations [3,4].

Treatment strategies for ABCs vary depending on lesion size, location, and proximity to critical structures such as the growth plate [4]. The mainstay of treatment is intralesional curettage with bone grafting, which preserves the physeal integrity while eliminating the lesion. Other treatment options include percutaneous sclerotherapy, arterial embolization, and minimally invasive techniques aimed at reducing recurrence while minimizing surgical morbidity [5]. In cases of extensive bone loss, more complex reconstructive techniques, such as bone transport or the Masquelet technique, may be considered [6,7]. However, the choice of treatment must be carefully tailored to the patient’s age, skeletal maturity, and the risk of complications such as growth disturbances, deformity, and recurrence.

In this case, the decision to perform an en bloc resection of the tumor resulted in physeal damage and progressive deformity, raising concerns about its appropriateness in such cases.

## Case presentation

We present a case of a three-year-old male patient who presented to our clinic following a low-energy trauma. The patient was playing and fell on the ground, subsequently developing pain and an inability to bear weight on his right leg. The patient did not report any previous pain. On examination, he had tenderness on palpation of the right distal thigh. The neurovascular exam was normal.

**Figure 1:**
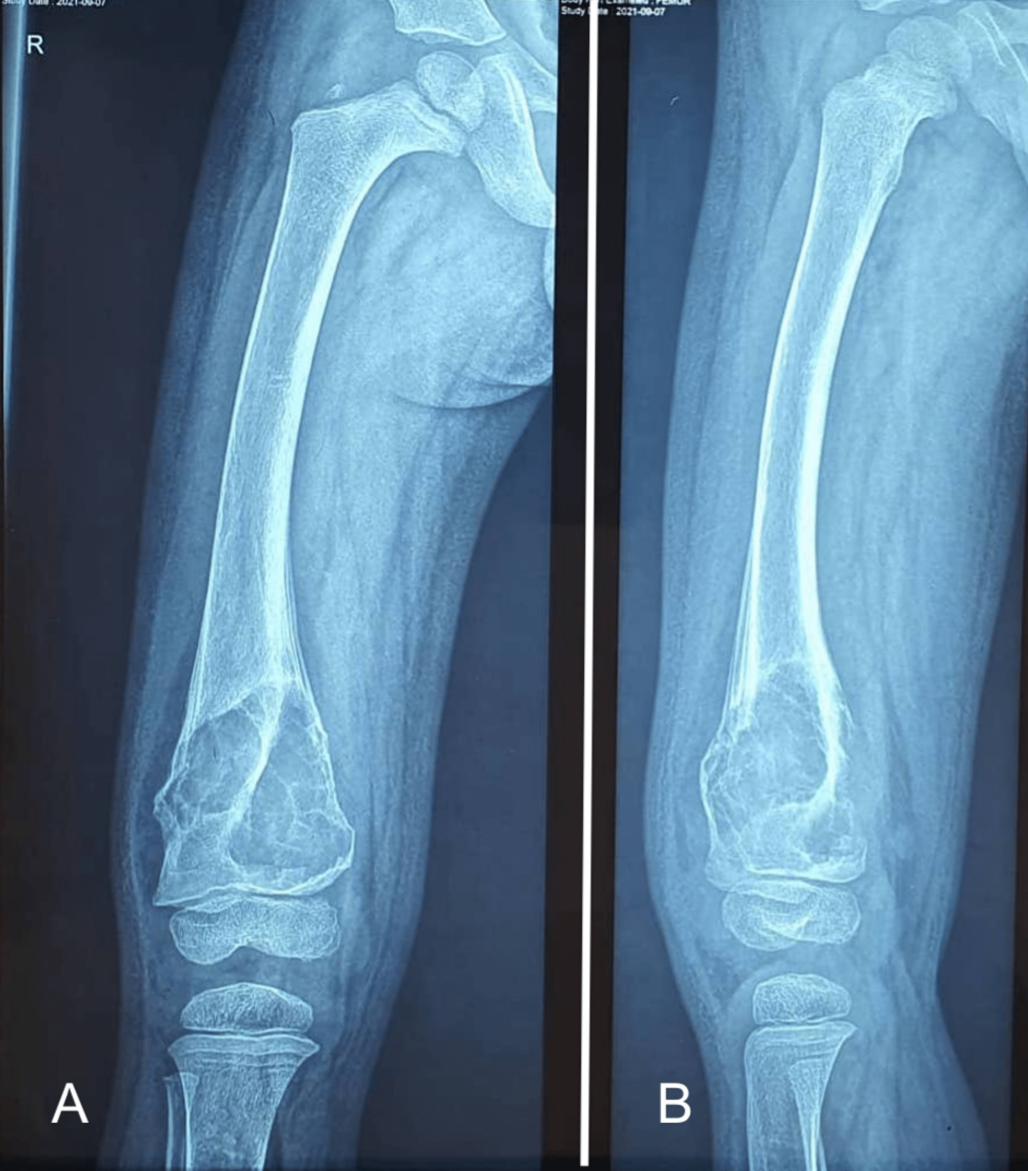
Plain radiographs of the right femur showing a lytic lesion of the distal femur. (A) AP view; (B) Lateral view.

Plain radiographs revealed a pathological fracture of the distal femur, with a tumor appearing as a borderline/benign osteolytic lesion above the distal femur physis (Figure 1); the lesion was well-defined, expansile, lytic with a thin, expanded, and scalloped cortical shell. The lesion exhibited internal septations.

**Figure 2:**
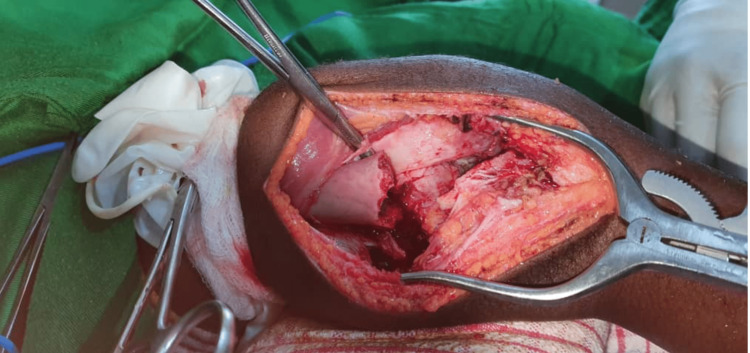
En bloc resection of the tumor.

**Figure 3:**
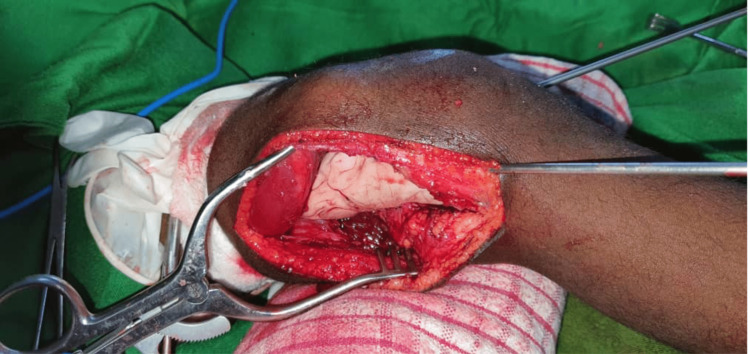
Bone cement placement with pin fixation.

**Figure 4:**
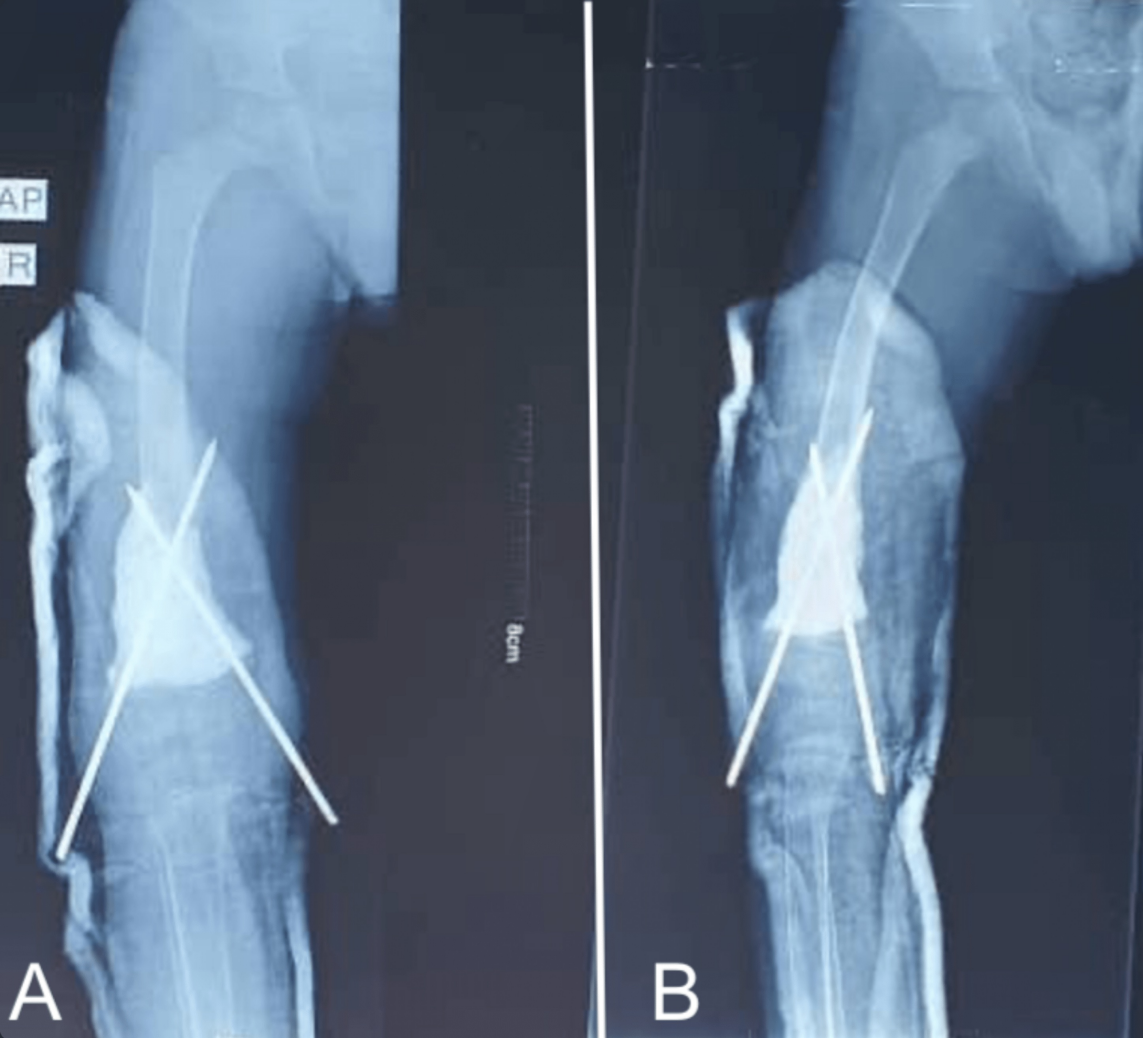
Postoperative X-ray following the first stage of the Masquelet technique. Bone cement placement with crossed pins for surgical fixation. The pin going to the medial side is too proud. (A) AP view; (B) Lateral view.

Based on these clinical and radiological findings, the decision was made to perform an en bloc resection of the tumor, which was sent for histopathology examination. The resection of the tumor left a 5 cm bone defect (Figure 2), which was filled with bone cement and stabilized with two smooth pins (Figure 3), and a long leg cast was applied (Figure 4) and maintained for three months.

**Figure 5:**
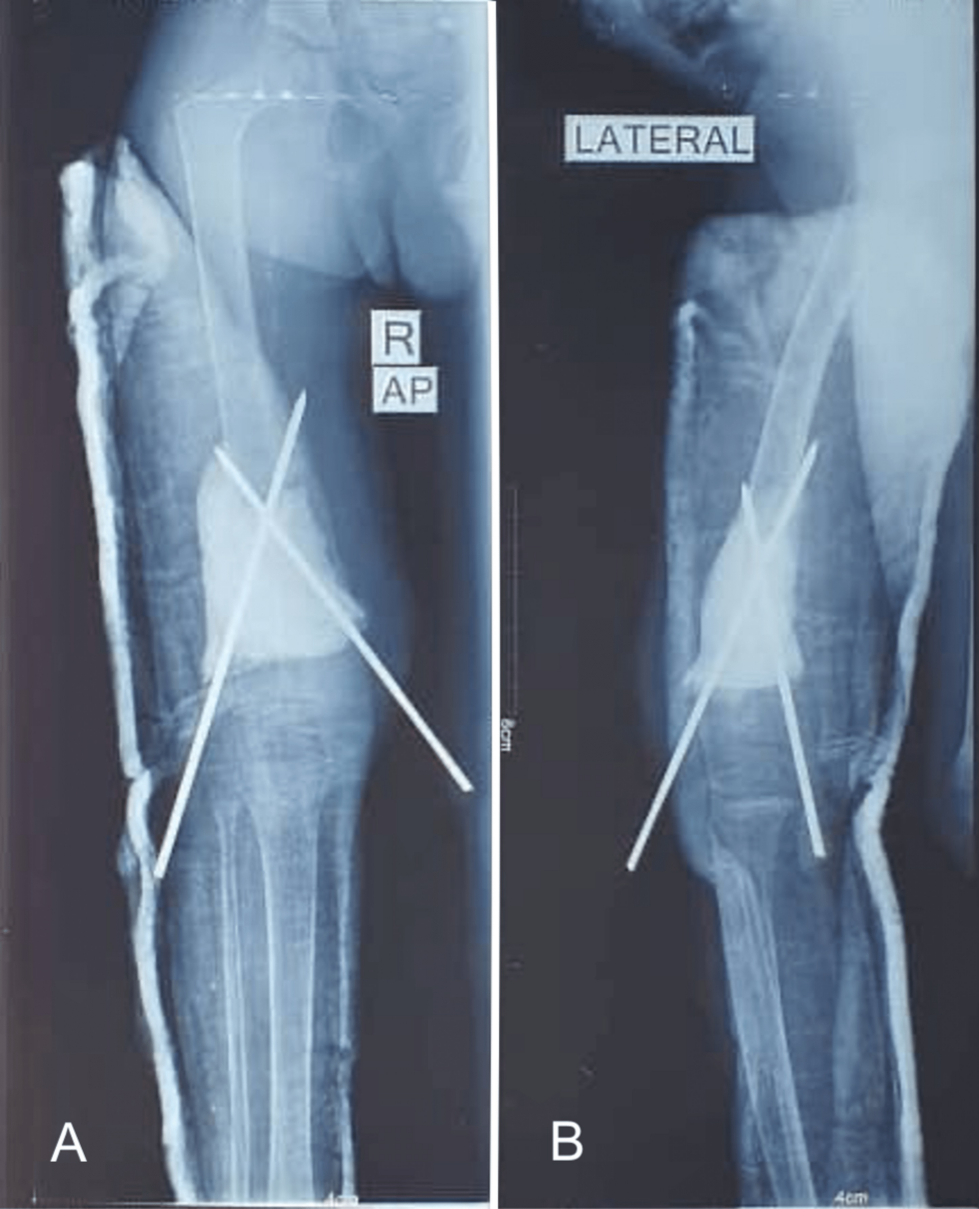
Six weeks following the first stage, bone growth can be seen medially on top of the cement. Pins were left in place, risking vascular injury. (A) AP view; (B) Lateral view.

**Figure 6:**
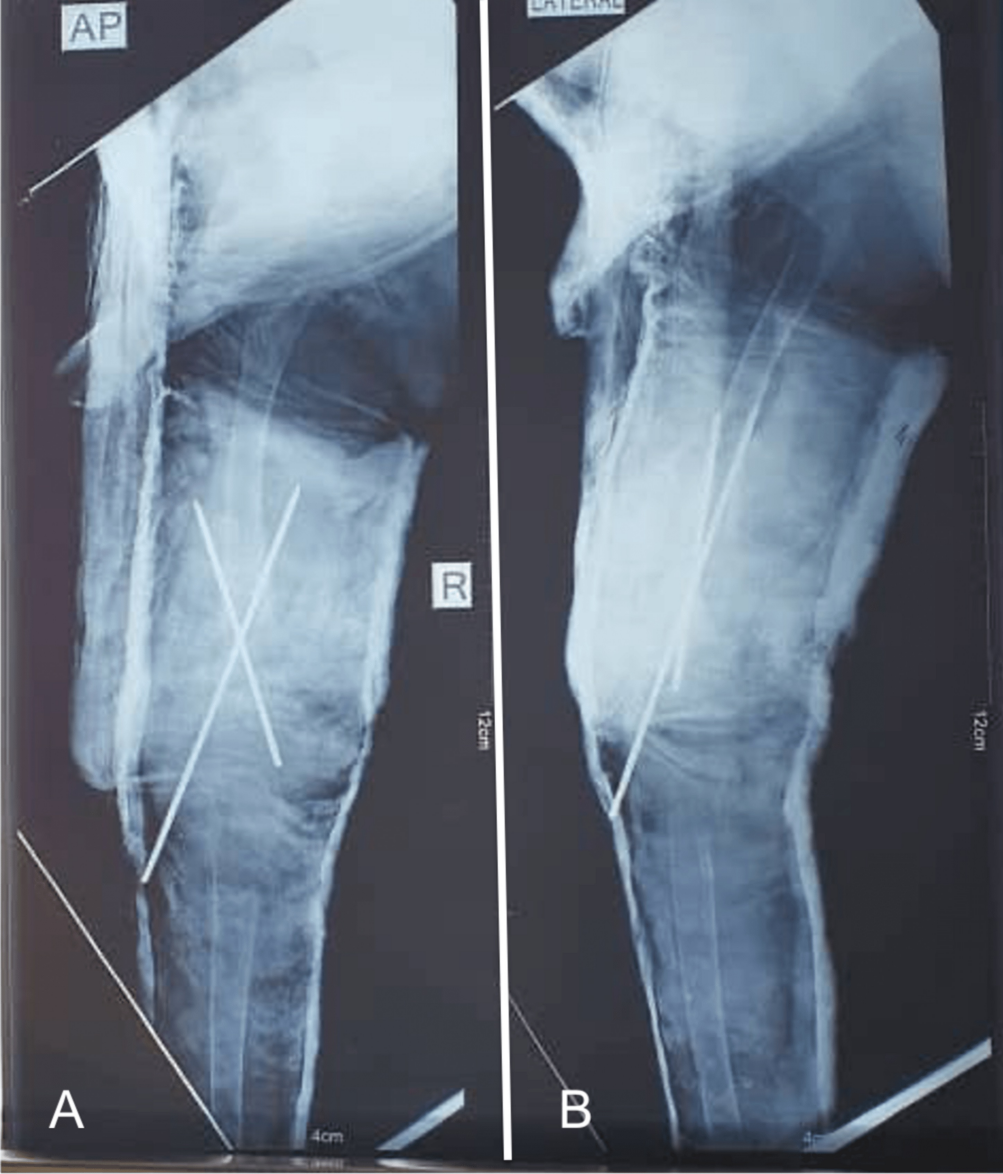
Postoperative X-ray following the second stage of the Masquelet technique. Pins still in place with dangerous advancement on the medial side. (A) AP view; (B) Lateral view.

Two weeks after the surgery, the pathology report confirmed the diagnosis of an ABC) tumor. We proceeded with the second stage of the Masquelet technique 6 weeks later, using a homolateral non-vascularized fibula graft and proximal tibia cancellous graft (Figures 5-6).

**Figure 7:**
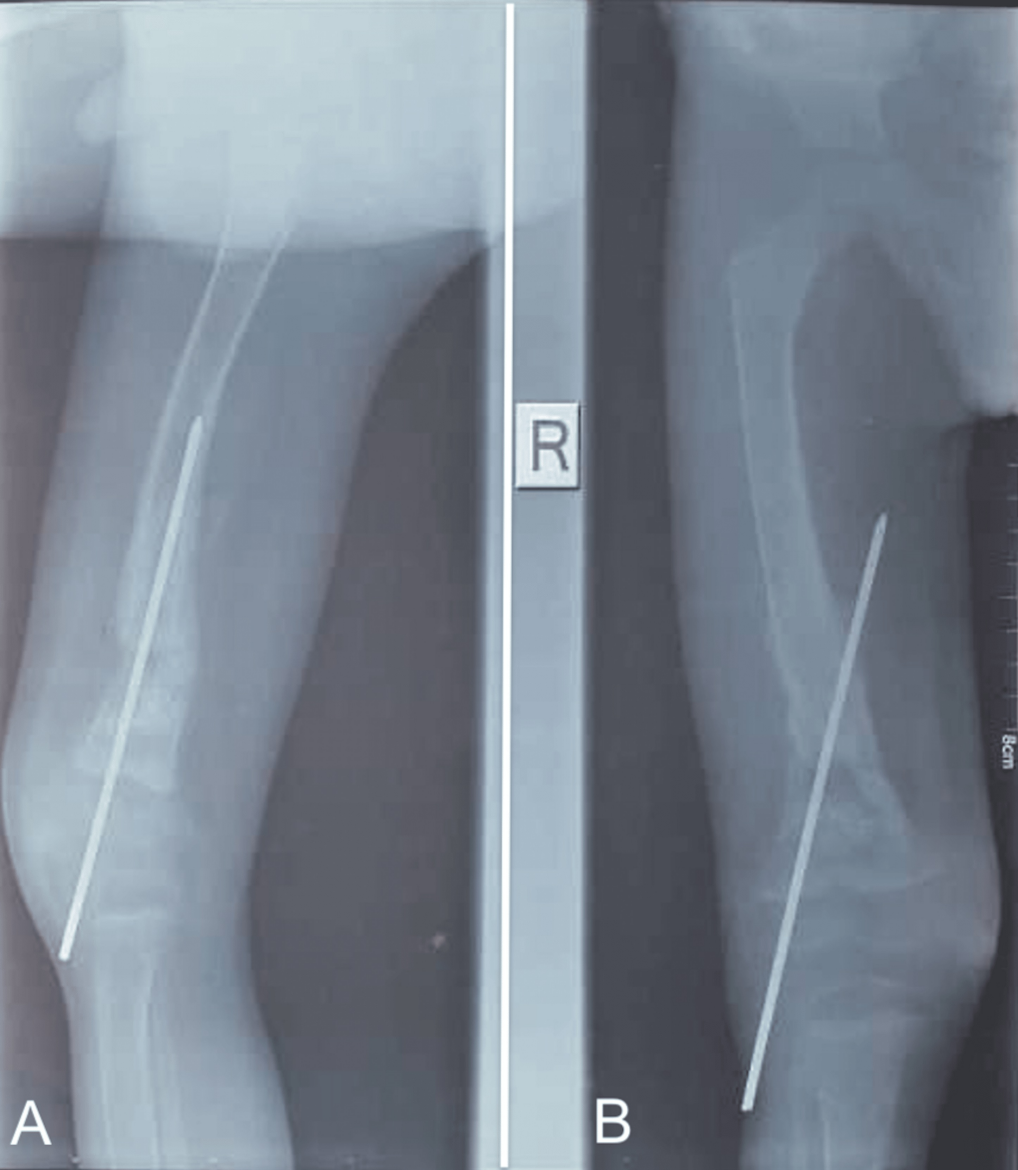
Healing at three months following the first surgery. The pin going towards the medial side is still in place and dangerously proud. (A) Lateral view; (B) AP view.

**Figure 8:**
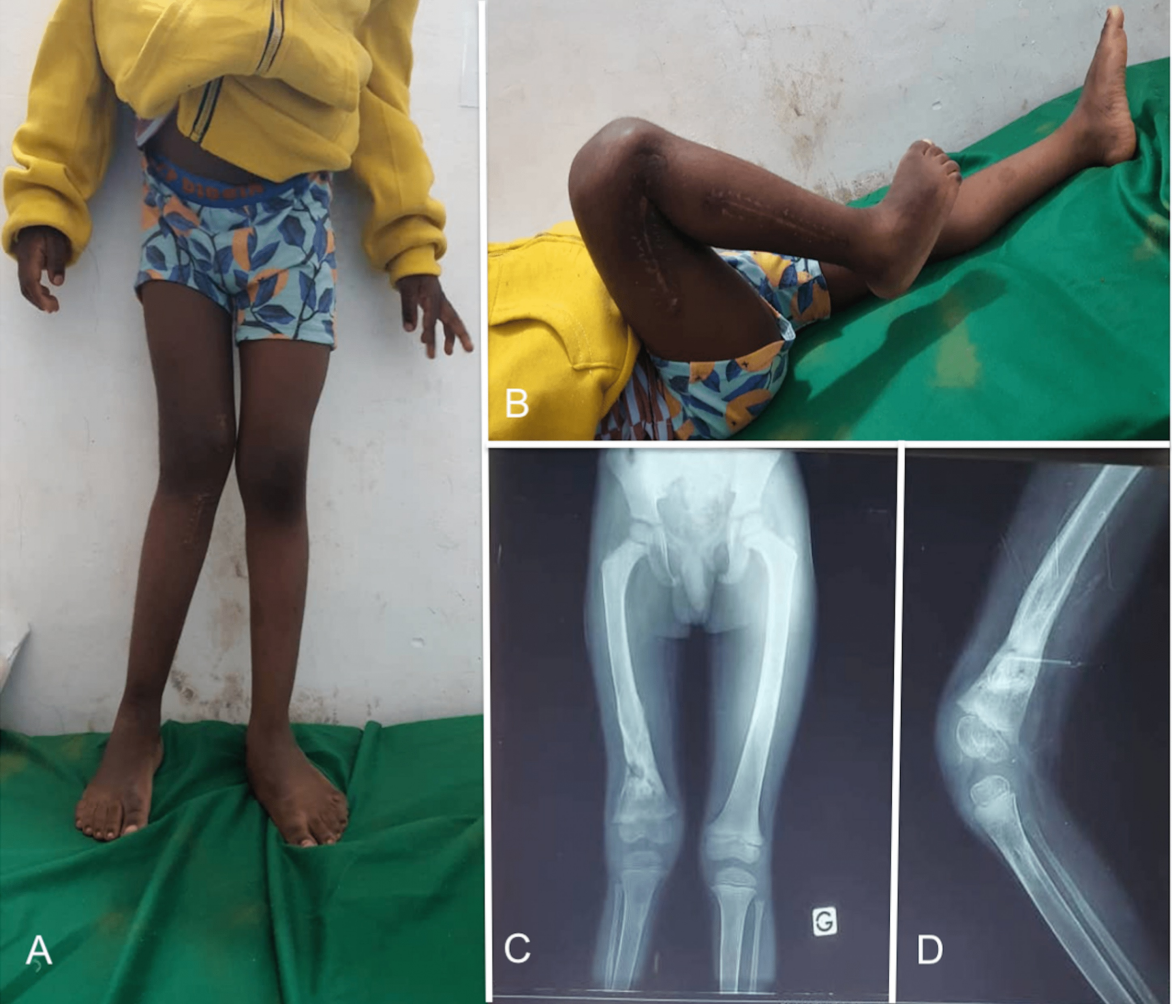
Follow-up at one year. (A) 2 cm of shortening, 20 degrees of knee valgus; (B) Full range of motion of the knee; (C, D) Complete integration of the graft.

At follow-up visits, the graft was incorporated at three months (Figure 7), and no local recurrence was detected at one year. Functionally, the patient could ambulate well, bear weight painlessly, and had a full range of motion of the knee (Figure 8) and hip. However, he had significant complications, including a 20-degree knee valgus and 2 cm limb length discrepancy, requiring further surgical intervention.

## Discussion

ABCs are locally destructive bone lesions characterized by blood-filled septate cavities lined by fibroblasts and histiocytes [3]. They commonly occur in the metaphyseal or metaphyseo-diaphyseal parts of long bones, with the proximal humerus, distal femur, proximal tibia, and spine being common locations [3,8]. In this case, the patient presented with a distal femur pathological fracture following low-energy trauma, which is a common initial presentation. The distal femoral metaphysis is a crucial weight-bearing location, and lesions in this region present unique diagnostic and therapeutic challenges.

ABCs have an incidence of approximately 0.14 per 100,000 persons per year and are most commonly diagnosed in adolescents. However, cases in younger children, such as this one, remain a minority [2,8].

The radiological features of ABCs typically include an eccentric, lytic, and expansile lesion with fine-walled cystic cavities and well-defined internal contours on plain radiographs [3]. In 1985, Capanna R et al. [9] proposed a very useful classification according to the tumor morphology that could be applied to the radiographic and gross specimen appearance. This classification divided ABC into five morphological subgroups. Per the Capanna classification, our case exemplifies a type 2 ABC. CT can provide additional information in complex anatomical regions, while MRI is the preferred modality for evaluating the lesion's extent and its relationship to the physis.

While MRI was not performed in this case due to financial constraints, it could have provided crucial insights into the lesion’s boundaries and proximity to the physis. This might have influenced the surgical approach, potentially leading to a more conservative, physeal-sparing technique such as curettage with bone grafting.

The definitive diagnosis of ABC is established through histopathological examination, identifying characteristic blood-filled cavities lined by fibroblasts and histiocytes [4]. In this case, the pathology report was only available postoperatively, highlighting a significant challenge in resource-limited settings, the lack of access to intraoperative frozen section analysis and, in some cases, the inability to perform preoperative biopsies. This limitation complicates the management of bone and soft tissue tumors, often forcing surgeons to make intraoperative treatment decisions without a confirmed diagnosis.

Standard management for type 2 ABCs involves intralesional curettage with bone grafting. However, in this case, curettage was not performed, likely due to the absence of preoperative MRI and intraoperative histopathological assessment, limitations frequently encountered in resource-limited settings.

Accurate diagnosis is critical for appropriate treatment selection, particularly to distinguish ABCs from telangiectatic osteosarcoma (TOC). TOCs are malignant, highly vascularized bone tumors that can mimic ABCs radiologically, as both present with multiple cystic compartments and fluid-fluid levels on imaging [10,11]. Given the diagnostic uncertainty, the surgical team opted for fixation and intraoperative assessment of the lesion. During surgery, the decision was made to proceed with en bloc resection and subsequent reconstruction using the Masquelet technique. While this approach successfully reconstructed the bone defect, it resulted in physeal damage, leading to valgus deformity and limb shortening.

A range of treatment modalities exist for ABCs, including medical therapy, curettage with bone grafting, and, in select cases, wide resection. Schreuder HW et al. reported that wide resection has a 0% recurrence rate, compared to approximately 31% for curettage and grafting. However, wide resection is associated with greater morbidity and the need for extensive reconstruction [4,12]. In resource-limited settings, recurrence management is challenging, often leading to more aggressive initial treatment strategies. This was demonstrated in the present case, where en bloc resection was favored over curettage to reduce the risk of recurrence. Unfortunately, this decision led to complications that could have been mitigated with a more physeal-sparing approach.

The challenge remains in balancing optimal treatment strategies with the realities of limited resources. While many case reports highlight successful outcomes, it is equally important to document suboptimal results to improve future clinical decision-making [13].

## Conclusions

This case highlights the importance of selecting age-appropriate treatment strategies for benign bone lesions in pediatric patients. While en bloc resection reduced the risk of recurrence, it introduced long-term complications, demonstrating the need for protocols that balance oncologic safety with functional outcomes, particularly in resource-limited settings. A more conservative approach, such as curettage with bone grafting, might have provided a better balance between local tumor control and physeal preservation. Proper preoperative imaging, meticulous surgical technique, and adherence to pediatric orthopedic principles are essential in preventing long-term deformities and ensuring optimal patient outcomes.
